# GPA peptide inhibits NLRP3 inflammasome activation to ameliorate colitis through AMPK pathway

**DOI:** 10.18632/aging.103825

**Published:** 2020-09-20

**Authors:** Zhao Deng, Jiangjin Ni, Xiaoyu Wu, Hongkui Wei, Jian Peng

**Affiliations:** 1 Department of Animal Nutrition and Feed Science, College of Animal Science and Technology, Huazhong Agricultural University, Wuhan 430070, Hubei, P. R. China; 2 The Cooperative Innovation Center for Sustainable Pig Production, Wuhan 430070, Hubei, China

**Keywords:** GPA, NLRP3, AMPK, ROS, colitis

## Abstract

Ulcerative colitis (UC) is a chronic and idiopathic inflammatory disease that affects the colon, resulting in immune dysregulation and the production of large amounts of pro-inflammatory cytokines. Pyroptosis and NLRP3 inflammasome are associated with various kinds of inflammatory diseases including colitis. The purpose of this study is to investigate the protective effects and regulatory mechanism of Gly-Pro-Ala (GPA) peptide on DSS-induced colitis. In vivo, we find GPA peptide could exert anti-inflammatory effects on DSS-induced mice colitis, and its anti-inflammatory effects are abolished in NLRP3^-/-^ mice. In macrophage, GPA suppresses the assembly of NLRP3 inflammasome and GSDMD cleavage. Furthermore, GPA maintains mitochondrial homeostasis through inhibiting ROS, mtDNA and NLRP3 mitochondrial localization, with other signals related to NLRP3 inflammasome unaffected. Furthermore, the inhibitory effects of GPA on reactive oxygen species (ROS) are found to be achieved by increasing AMPK phosphorylation. Our results suggest that GPA inhibits NLRP3 inflammasome activation through increasing AMPK phosphorylation to suppress ROS, and can be applied in the prevention of colitis through targeting NLRP3.

## INTRODUCTION

Ulcerative colitis (UC) is a chronic and idiopathic inflammatory disease that affects colon, resulting in weight loss, diarrhea, rectal bleeding, and abdominal pain [1, 2]. Under UC, microbes migrate from the lumen into the lamina propria, macrophages engulf microbes and send out inflammatory signals, producing large amounts of pro-inflammatory cytokines, resulting in a cycle of uncontrolled inflammation [3]. Macrophages, the gatekeepers of intestinal immune homeostasis, have been considered as a novel potential target to treat colitis [4].

Pyroptosis, an inflammatory cell death, can lead to cell membrane rupture, then ends to the release of cell contents and large number of inflammatory factors, such as IL-1β and IL-18 [5]. In the intestine, pyroptosis plays a central role to maintain gut homeostasis. Pyroptosis activation coordinates mucosal immune to defend intestines from infection, resulting in infected epithelial cells extrusion, and the fact that infected epithelial cells or macrophages being swallowed by neutrophils [6, 7]. Meanwhile, pyroptosis is overactivated in macrophages, it results in the production of a large number of pro-inflammatory cytokines, followed by the activation of immune cells in the lamina propria, and the disruption of the intestinal epithelial structure and intestinal homeostasis [3, 4]. Therefore, it is our concern whether controlling the occurrence of pyroptosis in macrophages can be an effective way to alleviate inflammatory response and maintain intestinal homeostasis.

Reactive oxygen species (ROS) are chemically active molecules containing oxygen, as the natural by-products of normal oxygen metabolism in vivo [8]. In colon tissues, ROS are mainly derived from mitochondria in epithelial cells and phagocytic cells (i.e macrophages and neutrophils) [9]. In gut homeostasis, ROS act as a lethal weapon in the defense of intestinal epithelium, for instance, in innate immunity, ROS are involved in respiratory bursts of phagocytes that kill incoming pathogens [10, 11]. However, in an intestinal pathological condition, ROS, as signal molecules, activate the inflammatory pathway to amplify inflammatory response and damage the intestinal structure [12]. Meanwhile, in intestinal macrophages, ROS, as a molecular signal, activate the NLRP3 inflammasome and secrete IL-1β and IL-18, resulting in pyroptosis and amplification of inflammatory response [13, 14]. Therefore, the relief of ROS production may be an important step to inhibit pyroptosis and treat colitis [3, 15].

AMP-activated protein kinase (AMPK) plays a key role in the regulation of energy homeostasis [16], and in inflammatory and oxidative stress [17, 18]. In our study, we found that GPA peptide, isolated from fish skin gelatin hydrolysate [19, 20], could ameliorate DSS-induced colitis by targeted inhibition of NLRP3 inflammasome-induced pyroptosis. Furthermore, GPA could increase AMPK phosphorylation to suppress ROS production and block NLRP3 mitochondrial localization, resulting in inhibiting the activation of NLRP3 inflammasome.

## RESULTS

### GPA inhibited NLRP3 inflammasome-induced pyroptotic cell death in macrophages

**Figure 1:**
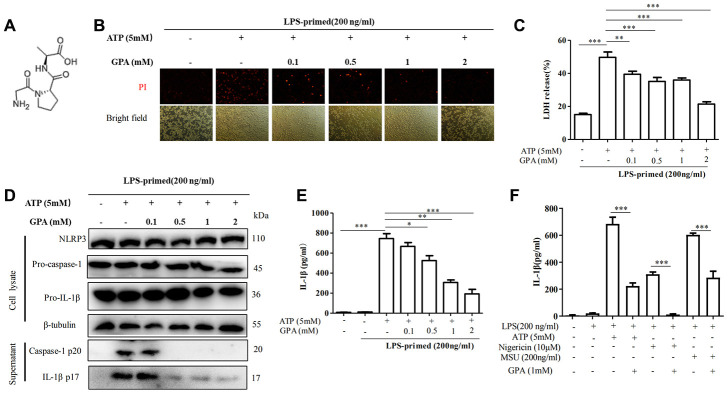
**GPA inhibited NLRP3 inflammasome-induced pyroptotic cell death in macrophages.** Chemical structure of GPA (**A**). THP-1 cells were primed with LPS for 4 h, followed by GPA treatment 6 h before stimulation with ATP for 30 minutes. cell death was measured by staining with propidium iodide (PI) (**B**), and LDH released (**C**). Immunoblot analyzed of IL-1β and caspase-1 in supernatants and cell lysate of THP-1 cells (**D**). IL-1β in supernatants of THP-1 cells was detected by ELISA (**E**). THP-1 cells were primed with LPS, followed by GPA treatment 6 h before stimulation with ATP, Nigericin or MSU, IL-1β in supernatants of THP-1 cells was detected by ELISA (**F**). Data are presented as mean ± SD, three independent experiments. ^*^p < 0.05, ^**^p < 0.01 and ^***^p < 0.001.

To test the effects of GPA on pyroptotic cell death and NLRP3 activation, we selected the optimal concentration in vitro according to the previous similar study [21], then examined the impact of GPA (Figure 1A) on cell death, caspase-1 activation and IL-1β secretion. We treated lipopolysaccharide (LPS)-primed in THP-1 cells with GPA before ATP stimulation. We found GPA inhibited the formation of pyroptotic pores, as measured by propidium iodide (PI) uptake, and cell death, as measured by lactate dehydrogenase (LDH) release (Figure 1B, 1C). Subsequently, we examined the effects of GPA on NLRP3 inflammation activation, through measuring caspase-1 cleavage and IL-1β secretion. The results showed that GPA treatment blocked ATP-induced the release of activated caspase-1 p20 and mature IL-1β into the culture supernatants, without affecting the level of NLRP3, pro-caspase-1 and pro-IL-1β (Figure 1D, 1E). Further, we tested whether GPA could suppress NLRP3 inflammasome activated by other NLRP3 activators, i.e Nigericin and MSU, we found GPA significantly inhibited ATP, Nigericin or MSU-induced IL-1β recreation (Figure 1F). Meanwhile, GPA treatment inhibited ATP-induced the release of activated caspase-1 p20 and mature IL-1β into the culture supernatants in BMDMs (Supplementary Figure 1A). Furthermore, we compared the anti-inflammatory effects between GPA, MCC950 (NLRP3 antagonists), and amino acids. The results showed that GPA, MCC950, and Glycine inhibited LDH released in THP-1 cells (Supplementary Figure 1B), and GPA and MCC950 inhibited IL-1β released in THP-1 cells (Supplementary Figure 1C). Further, GPA significantly inhibited cLPS-induced non-canonical NLRP3 inflammasome activation, as measured LDH released, caspase-1 cleavage and IL-1β secretion (Supplementary Figure 2A–2C). Taken together, these results indicated that GPA could inhibit NLRP3 inflammasome activation and pyroptosis.

### GPA relieved DSS-induced colitis in a NLRP3-dependent manner

To determine the anti-inflammatory effect of GPA *in vivo*, we examined its anti-inflammatory effects on a mice model of colitis induced by DSS. Then we detected the level of IL-1β and LDH in serum, caspase-1 p20 and mature IL-1β in colon tissues. The results showed that the supplementation of GPA at doses of 50, 100 and 150 mg/kg body weight significantly inhibited DSS-induced IL-1β secreted, caspase-1 activated and GSDMD cleavage (Supplementary Figure 3A, 3B). Furthermore, GPA suppressed pyroptotic cell death through detected LDH in serum (Supplementary Figure 3C). These results indicated that GPA alleviated DSS-induced colitis through suppressing caspase-1 cleavage and IL-1β secretion.

**Figure 2:**
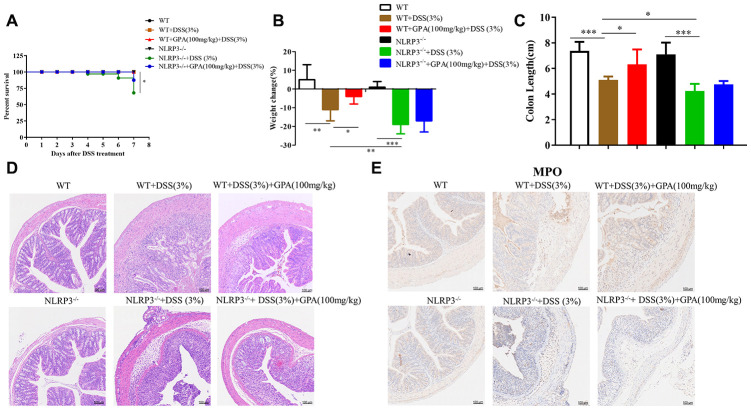
**GPA relieved DSS-induced colitis in a NLRP3-dependent manner.** Wild-type (WT) and NLRP3-/- mice were treated with 3% DSS in their drinking water for 7 days to induce acute colitis. GPA (100mg/kg) was administered for 14 days before and during DSS treatment *via* oral gavage once per day. Mice were sacrifced at day 14 (n=10 mice/group). Percentage survival of mice after DSS treatment (**A**). Weight change of mice during the experiment (**B**). The lengths of colons from each group of mice were measured (**C**). H&E stains of serial sections of colons, histopathological scores of each group were determined (**D**). Myeloperoxidase (MPO) activity in the colonic tissues was detected (**E**). Data are presented as mean ± SD, n=10 /group. ^*^p < 0.05, ^**^p < 0.01 and ^***^p < 0.001.

Next, to determine the role of NLRP3 in the anti-inflammatory effects of GPA, we constructed *NLRP3^-/-^* mice to further determine whether the anti-inflammatory effects of GPA depended on NLRP3. Again, the administration of GPA (100 mg/kg) resulted in a significant alleviation of DSS-induced colitis in wild-type mice. However, in *NLRP3^-/-^* mice, GPA failed to ameliorate DSS-induced morbidity, weight loss, colon shortens, crypt damage, inflammation and spleen hypertrophy (Figure 2A–2D, Supplementary Figures 4A–4C and 5A, 5B). These results showed that the anti-colitis effect of GPA was depleted in *NLRP3^-/-^* mice. Furthermore, we found GPA treatment contributed to the maintenance of the integrity of the intestinal structure, and inhibited MPO hyperactivity in wild-type mice (Figure 2D, 2E and Supplementary Figure 4C, 4D), but the protective effects were abolished in *NLRP3^-/-^* mice. We found that tight junction was aggravated destruction in *NLRP3^-/-^* mice, leading to an increase in the destruction of intestinal structures and recruitment of neutrophils (Figure 2D and Supplementary Figure 5C). Overall, these results suggested that NLRP3 expression is critical for protection against DSS-induced colitis by GPA.

### GPA inhibited NLRP3 inflammasome assembly and GSDMD cleavage

**Figure 3:**
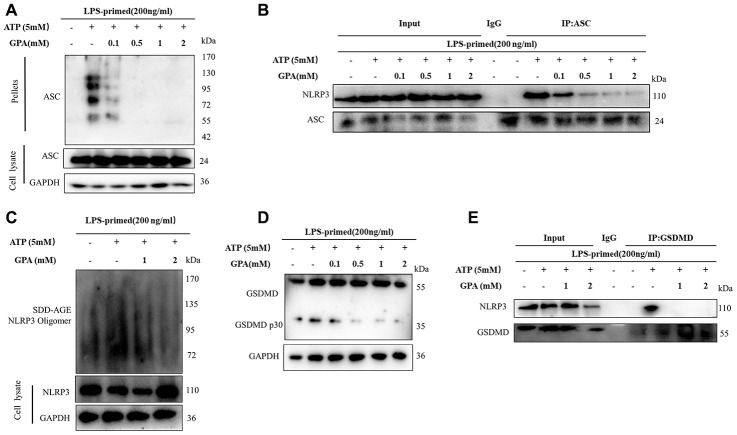
**GPA inhibited NLRP3 inflammasome assembly and GSDMD cleavage.** THP-1 cells were primed with LPS for 4h, followed by GPA treatment 6 h before stimulation with ATP for 30 min. Immunoblot analyzed of ASC oligomerization in lysates of THP-1 cells (**A**). IP and immunoblot analyzed of the interaction of endogenous NLRP3 and ASC in THP-1 cells (**B**). Immunoblot analyzed of NLRP3 by SDD-AGE assay in THP-1 cells (**C**). Immunoblot analyzed of cleavage of GSDMD in THP-1 cells (**D**). IP and immunoblot analyzed of the interaction of endogenous NLRP3 and GSDMD in THP-1 cells (**E**). Three independent experiments.

Next, we explored the mechanism of how GPA blocked NLRP3 activation. Firstly, we found that GPA could suppress ATP-induced ASC oligomerization (Figure 3A). And another critical step for NLRP3 activation is the recruitment of ASC to NLRP3 [13]. The effect of GPA on the endogenous NLRP3 oligomerization was confirmed through using semi-denaturing detergent agarose gel electrophoresis (SDD-AGE) (Figure 3B), a method for detecting large protein oligomers in studying prions. Moreover, we determined whether GPA could inhibit NLRP3–ASC interaction, and the results showed that GPA blocked the endogenous interaction between NLRP3 and ASC in ATP-treated macrophages (Figure 3C and Supplementary Figure 6A).

GSDMD is an executor of pyroptosis and required for IL-1β secretion, then we examined the effects of GPA on GSDMD cleavage [22]. We found GPA treatment suppressed the ATP-induced release of activated GSDMD p30 (Figure 3D). Furthermore, GPA inhibited the endogenous interaction between GSDMD and NLRP3 and GSDMD oligomerization in ATP-treated macrophages (Figure 3E and Supplementary Figure 6B). These results indicated that GPA could inhibit pyroptotic cell death by blocking NLRP3 oligomerization and GSDMD cleavage.

### GPA reduced ROS and mtDNA production and suppressed NLRP3 mitochondrial localization

**Figure 4:**
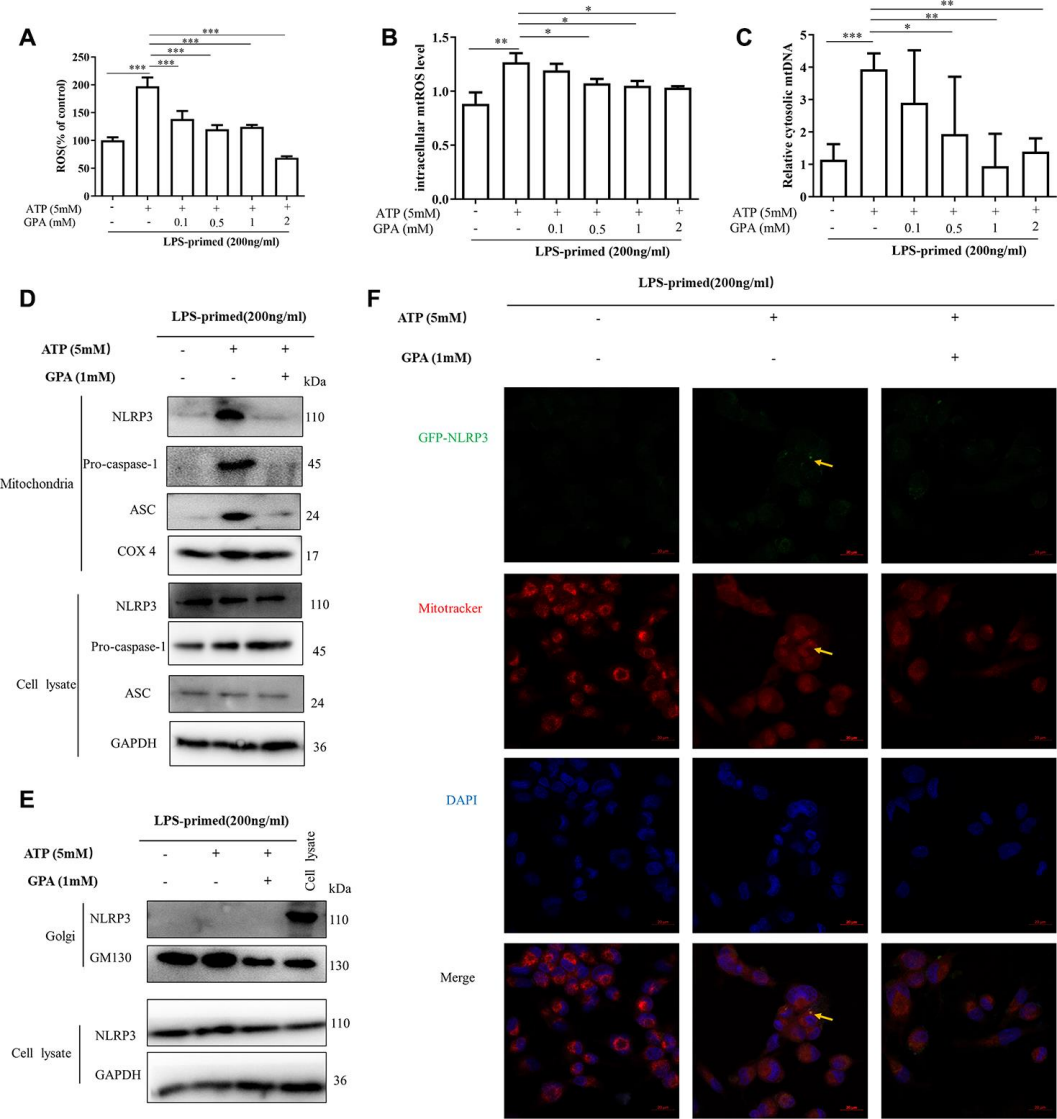
**GPA reduced ROS and mtDNA production, and suppressed NLRP3 mitochondrial localization.** THP-1 cells were primed with LPS for 4h, followed by GPA treatment 6 h before stimulation with ATP for 30 min. Levels of the ROS and mtROS were measured in THP-1 cells (**A**, **B**). Quantitative real-time PCR analyzed of mtDNA in THP-1 cells (**C**). Immunoblot analyzed of mitochondrial components of NLRP3 inflammasome in THP-1 cells through extract mitochondrial (**D**). Immunoblot analyzed of Golgi components of NLRP3 inflammasome in THP-1 cells through extract Golgi (**E**). Immunofluorescence analyzed of mitochondrial components of NLRP3 in THP-1 cells (**F**). Data are presented as mean ± SD, three independent experiments, ^*^p < 0.05, ^**^p < 0.01 and ^***^p < 0.001.

As described above, we found that GPA significantly suppressed the oligomerization of ASC and NLRP3 (Figure 3). It suggested that GPA may act upstream of NLRP3 to inhibit NLRP3 inflammasome activation. We then studied whether GPA could directly bind to NLRP3 inflammasome, or affect cell signaling related to NLRP3 inflammasome activation, i.e potassium efflux, chloridion efflux, calcium afflux, ROS and mtDNA production. Unfortunately, we found that GPA could not directly bind with any protein related to NLRP3 (Supplementary Figure 7A). For cell signaling about NLRP3 activation, we found GPA treatment significantly suppressed the release of ROS, mtROS, and mtDNA, without affecting potassium, chloridion and calcium (Figure 4A–4C and Supplementary Figure 8A–8C). And in DSS-induced colitis, GPA treatment significantly decreased H_2_O_2_ production in serum (Supplementary Figure 8D).

ROS and mtDNA production are related to mitochondrial homeostasis, and mitochondria play a critical role in NLRP3 inflammasome [13]. Resting NLRP3 localizes to endoplasmic reticulum structures, whereas on inflammasome activation, NLRP3 redistributes to mitochondria or Golgi [23, 24]. Subsequently, we examined whether GPA affected the co-localization of NLRP3, through extracted organelle protein and immunofluorescence. Interesting, we found that ATP treatment promoted NLRP3, ASC, caspase-1 translocated into mitochondria, not Golgi, in THP-1 cells by extracting mitochondria and Golgi (Figure 4D, 4E), then we confirmed the results by immunofluorescence (Figure 4F and Supplementary Figure 9A). And GPA treatment inhibited NLRP3 co-localize in mitochondria (Figure 4D, 4E). The results demonstrated that after NLRP3 inflammation activation, NLRP3 translocates into mitochondria not Golgi in THP-1 cells, and GPA suppresses NLRP3 co-localize with mitochondria, and inhibits ROS and mtDNA production.

### GPA blocked NLRP3 activation through inhibiting ROS production

**Figure 5:**
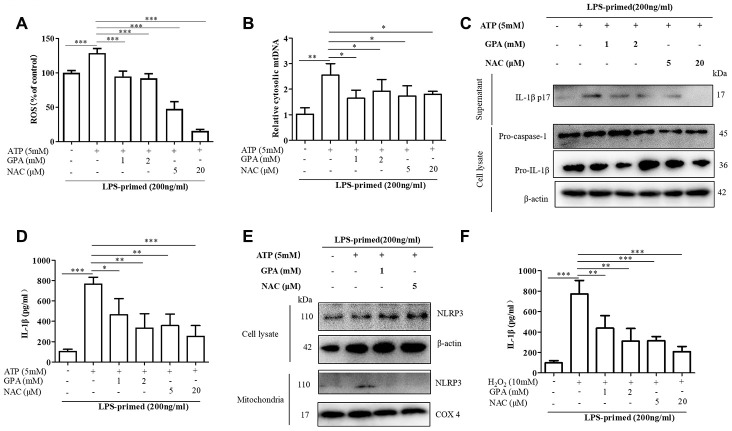
**GPA blocked NLRP3 activation by inhibiting ROS production.** THP-1 cells were primed with LPS for 4h, followed by GPA or NAC treatment 6 h before stimulation with ATP for 30 min. Levels of the ROS was measured in THP-1 cells (**A**). Quantitative real-time PCR analyzed of mtDNA in THP-1 cells (**B**). Immunoblot analyzed of IL-1β in supernatants and cell lysate of THP-1 cells (**C**). IL-1β in supernatants of THP-1 cells was detected by ELISA (**D**). Immunoblot analyzed of mitochondrial components of NLRP3 inflammasome in THP-1 cells (**E**). THP-1 cells were primed with LPS for 4h, followed by GPA treatment 6 h before stimulation with H_2_O_2_ for 4 h, IL-1β in supernatants of THP-1 cells was detected by ELISA (**F**). Data are presented as mean ± SD, three independent experiments. ^*^p < 0.05, ^**^p < 0.01 and ^***^p < 0.001.

To explore whether GPA suppressed ROS production to maintain mitochondrial homeostasis and inhibit NLRP3 activation. We set NAC, ROS scavenger, as a positive control group. We found GPA or NAC treatment suppressed ROS production and mtDNA secretion (Figure 5A, 5B). Moreover, GPA or NAC treatment inhibited ATP-induced the release of mature IL-1β into the culture supernatants in THP-1 cells (Figure 5C, 5D). Subsequently, we examined whether suppressed ROS production could affect the co-localization of NLRP3. The results showed that GPA or NAC treatment inhibited NLRP3 co-localize in mitochondria (Figure 5E).

Additional support for the hypothesis that GPA inhibits NLRP3 inflammasome by reducing ROS was provided by experiments using H_2_O_2_, which resulted in more mature IL-1β secretion in THP-1 cells (Figure 5F). And the generation of ROS, increased by H_2_O_2_ was suppressed by GPA (Supplementary Figure 10A). Similarly, GPA reduced expression of IL-1β in the supernatants in response to H_2_O_2_ (Figure 5F). These results suggested that GPA could inhibit NLRP3 activation by suppressing ROS production.

### AMPK phosphorylation mediated the effects of GPA on decreasing ROS production

**Figure 6:**
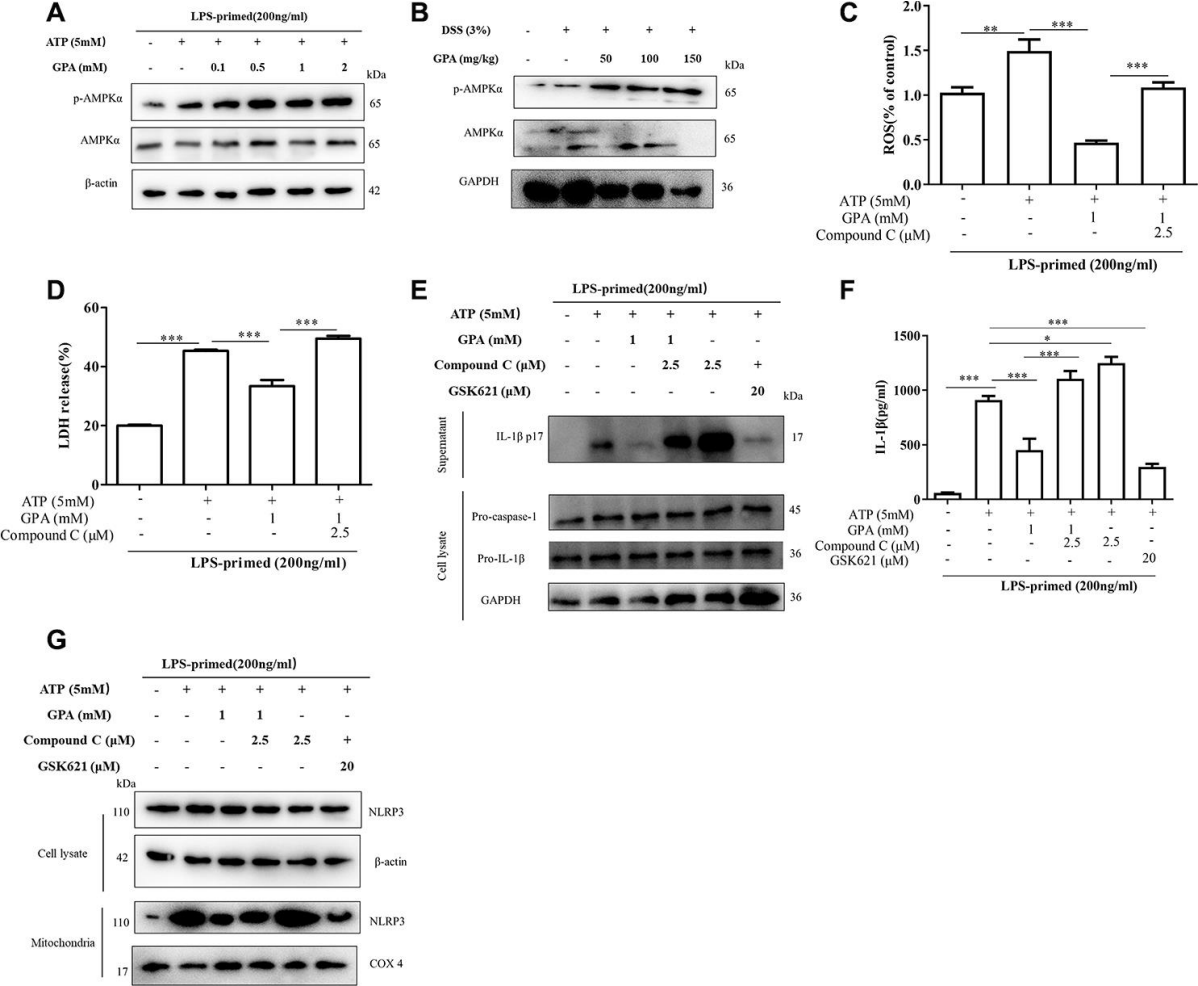
**AMPK phosphorylation mediated the effects of GPA on decreasing ROS production.** THP-1 cells were primed with LPS for 4 h, followed by GPA treatment 6 h before stimulation with ATP for 30 min, immunoblot analyzed of AMPK phosphorylation in cell lysate of THP-1 cells (**A**). Immunoblot analyzed of AMPK phosphorylation in colon tissues of mice (**B**). THP-1 cells were primed with LPS, followed by GPA, Compound C or GSK621 treatment 6 h before stimulation with ATP for 30 min. Levels of the ROS was measured in THP-1 cells (**C**). Cell death was measured by and LDH released (**D**). Immunoblot analyzed of IL-1β in supernatants and cell lysate of THP-1 cells (**E**). IL-1β in supernatants of THP-1 cells was detected by ELISA (**F**). Immunoblot analyzed of mitochondrial components of NLRP3 inflammasome in THP-1 cells (**G**). Data are presented as mean ± SD, three independent experiments. ^*^p < 0.05, ^**^p < 0.01 and ^***^p < 0.001.

Next to explore the mechanism of GPA on inhibiting ROS and NLRP3 activation, we examined signal pathways involved in removal of ROS. We found GPA treatment increased AMPK phosphorylation in THP-1 cells, without affecting LC3 and Nrf2 (Figure 6A and Supplementary Figure 11A, 11B), and we confirmed the results on mice colon tissues (Figure 6B). Subsequently, Compound C, an AMPK inhibitor, was used to confirm whether GPA could suppress ROS production through increasing AMPK phosphorylation. The results showed that the inhibitory effects of GPA on ROS and LDH were blocked through treated with Compound C (Figure 6C, 6D). For NLRP3 activation, we found that the effects of GPA on inhibiting ATP-induced the release of mature IL-1β into the culture supernatants were blocked by Compound C, and GSK621 (AMPK activator) inhibited mature IL-1β secretion, without affecting the level of pro-caspase-1 and pro-IL-1β (Figure 6E, 6F). Next, we found Compound C increased NLRP3 co-localize in mitochondria, and GSK621 decreased NLRP3 co-localize in mitochondria (Figure 6G). These results indicated that GPA could increase AMPK phosphorylation to suppress ROS production, resulting in inhibiting the activation of NLRP3 inflammasome.

## DISCUSSION

In the gut, intestinal macrophages, located in lamina propria, produce a variety of cytokines and other soluble factors, which help maintain intestinal homeostasis [4]. For instance, prostaglandin E2 (PGE2), which allows local macrophages to stimulate the proliferation of epithelial progenitor cells in the intestinal recess, thereby regulating the integrity of the epithelial barrier [25]. However, when intestinal homeostasis is disturbed, intestinal macrophages pool changes a lot [25]. For instance, in colitis, the structure of epithelium is disrupted, enabling microbes to migrate from the lumen into the lamina propria, macrophages engulf or touch microbes, leading to producing a lot of inflammatory cytokines and reactive oxygen species (ROS) [4, 26]. Hence, macrophages have become a potential therapeutic target in colitis, through blocking immune signals [3, 4]. In the current study, we found that GPA exerts anti-inflammatory effects in macrophages, and through *knockout NLRP3* in mice, the anti-inflammatory effects of GPA were found to target NLRP3. And we found that NLRP3 deficiency aggravates DSS-induced colitis by exacerbating gut structure and tight junction (Occludin). Because NLRP3 inflammasome protects against loss of epithelial integrity and mortality during experimental colitis [27]. However, the role of NLRP3 in colitis is controversial, several studies reported that NLRP3 deficiency exerts a protective effect in DSS induced colitis [28–30]. And the effects of GPA on level of GSDMD, pro-caspase-1 and pro-IL-1β are different *in vivo and in vitro*, it may due to other types of cells in the gut. These controversies may need further study.

Several molecular and cellular events have been proposed as the trigger for NLRP3 inflammasome activation, including K^+^ efflux, Cl^-^ efflux, Ca^2+^ signaling, ROS, mtDNA, and lysosomal rupture, but the precise mechanism of NLRP3 activation is still unclear [13]. To explore the key signal or mechanism of GPA inhibits NLRP3 activation, we found that GPA inhibits NLRP3 activation, through suppressing ROS and mtDNA, without affecting other signals related to NLRP3 inflammasome. In the gut, ROS is produced by the mitochondrial electron transport chain, endogenous oxidase pathway (including NADPH oxidase, NADPH oxidase isoforms (NOX), xanthine oxidase (XO), lipoxygenases (LOXs), glucose oxidase, myeloperoxidase (MPO), nitric oxide synthase, and cyclooxygenases (COXs) and exogenous way (intestinal flora, food, drug, toxin) [8]. As signaling molecules, ROS stimulate macrophages, resulting in producing a large number of inflammatory factors, and damaging the intestinal structure [31–33]. Therefore, the relief of ROS production becomes an effective way to block inflammatory response and treat colitis [3, 15]. Subsequently, in our study, we found GPA could suppress NLRP3 inflammasome activation through inhibiting ROS production *in vivo* and *in vitro*. Therefore, GPA treatment is an effective way to suppress ROS production and prevent colitis.

Mitochondrial, as the major site of ROS production in most mammalian cells, plays a central role in colitis and NLRP3 inflammasome activation [8, 23, 34]. Resting NLRP3 localizes to endoplasmic reticulum structures, whereas once NLRP3 inflammasome activation, both NLRP3 and its adaptor ASC co-localize with endoplasmic reticulum and mitochondria organelle clusters [23], while Chen reported that once NLRP3 inflammasome activation, NLRP3 co-localize with trans-Golgi, not mitochondria [24]. And in the present study, we found after ATP-induced NLRP3 inflammasome activation, NLRP3 translocates into mitochondria, not Golgi, and GPA treatment suppresses NLRP3 co-localize with mitochondria. The reason for the contradiction about NLRP3 co-localizes may be caused by cell types or ASC. In Chen’s research, once NLRP3 inflammasome activation, NLRP3 co-localize with trans-Golgi in HeLa or *ASC^-/-^* BMDM [24], ASC allows the assembly of inflammasomes to be extremely rapid, hindering the observation of changes in the upstream receptor NLRP3 after activation [35]. To our knowledge, this is the first report that nutrients or natural products suppress NLRP3 inflammasome activation through blocking NLRP3 co-localize with mitochondria.

AMPK, as an energy receptor, plays a central role in maintaining mitochondrial homeostasis, controlling inflammatory stress and oxidative stress [36, 37]. AMPK is considered as a potential target for metabolic disorders, including diabetes, obesity and fatty liver disease, cancer and colitis [37–39]. GPA peptide could be nutrients, which may provide energy for intestine through activates AMPK pathway. And in the current study, GPA suppresses ROS production through increasing phosphorylation of AMPK in macrophages. It provides that AMPK could be a target to control NLRP3 inflammasome activation and colitis.

**Figure 7:**
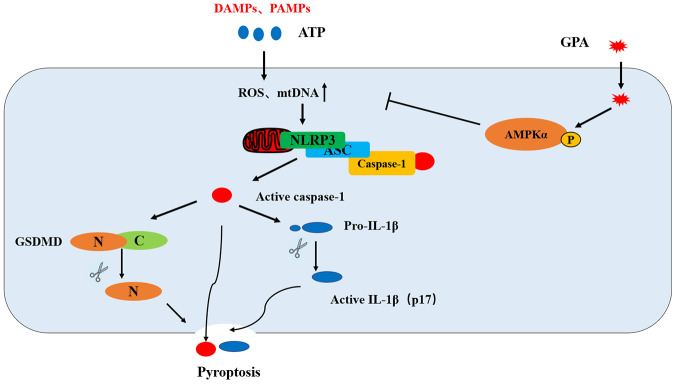
**Putative mechanism for the anti-inflammatory effect of GPA in macrophages.** GPA increased AMPK phosphorylation to suppress ROS and mtDNA production, resulting in the blocking of NLRP3 mitochondrial translocation, which inhibited NLRP3 inflammasome activation and pyroptosis.

In summary, GPA ameliorates DSS-induced colitis in a NLRP3-dependent manner. GPA increases the phosphorylation of AMPK, where it blocks ROS and mtDNA production as well as NLRP3 mitochondrial translocation, leading to suppressing NLRP3 inflammasome activation and pyroptosis in macrophages (Figure 7). Our results suggest that GPA could be potentially used for the prevention of colitis.

## MATERIALS AND METHODS

### Reagents and antibodies

Synthetic peptides GPA were purchased from Top-peptide Biotechnology Co., Ltd (Shanghai, China). Dextran sulfate sodium (DSS, molecular weight of 36–50 kDa) was purchased from MP Biomedicals (Irvine, CA, USA). LPS (L6230) was purchase from Sigma-Aldrich (St. Louis, MO, USA). Protease Inhibitor Cocktail (C0001) was purchased from Target Mol (Topscience, Shanghai, China). Compound C (S7306) GSK621 (S7898) were purchased from Selleck (Shanghai, China). NAC (A601127) was purchased from Sangon Biotech (Shanghai, China). Cell lysis buffer for Western analysis (P0013), phenylmethanesulfonyl fluoride (PMSF) (ST505) were purchased from Beyotime (Shanghai, China). DAPI (D4054) was purchased from US Everbright®Inc (Suzhou, China). The antibodies against NLRP3 (A5652), GSDMD (A18281), TOM20 (A19403), GM130 (A5344), LC3B (A11282), β-actin (AC026) were purchased from Abclonal (Wuhan, China). The antibodies against human IL-1β (85658) was obtained from Cell Signaling Technology (Danvers, USA). The antibodies against p-AMPK (AF3423) AMPK (AF6423) and mouse IL-1β (AF4006) were purchased from Affinity (Cincinnati, USA). The antibody against human caspase-1 (AG-20B-0048) and mouse caspase-1 (AG-20B-0042) were brought from Adipogen (AntGene, Wuhan, China). The antibody against ASC (sc-514414) was brought from Santa Cruz Biotechnology (Shanghai, China) The antibodies against GAPDH (GB1102) was purchased from Servicebio (Wuhan, China).

### Cell culture and stimulation

Human acute monocytic leukemia THP-1 cells were obtained from BeNa Culture Collection (Beijing, China) and were authenticated. THP-1 cells were cultured in RIPA 1640 (Gibco, San Diego, CA, USA) containing 10% fetal bovine serum (FBS, Gibco, San Diego, CA, USA) and 1% penicillin/streptomycin at 37°C under a 5% CO_2_ atmosphere. Differentiation of THP-1 cells were induced by 100nM phorbol 12-myristate 13-acetate (PMA) for 4 h.

Bone marrow macrophages were derived from C57BL/6 mice and cultured in DMEM complemented with 10% FBS, supplemented with 10 ng/ml M-CSF.

For induction of canonical NLRP3 inflammasome activation, 5 × 10^5^ macrophages were plated overnight in 12-well plates and the medium was changed to Opti-MEM (1% FBS) in the following morning, and then cells were treated with 200 ng/ml LPS for 4 h. After that, GPA was added into the cell culture for another 6 h, then the cells were stimulated for 4 h with MSU (200ng/ml), or for 30 min with ATP (5 mM) or nigericin (10 μM).

For induction of non-canonical NLRP3 inflammasome activation, 5 × 10^5^ macrophages were plated overnight in 12-well plates and the medium was changed to Opti-MEM (1% FBS) in the following morning, and then cells were treated with 400 ng/ml Pam3CSK4 (InvivoGen) for 4 h. After that, GPA was added into the cell culture for another 16 h, together cells were transfected with cytoplasm LPS (cLPS) (500ng/ml) for16 h. All cell tests were repeated three times (independent experiments), four samples at a time.

### Animals

Male C57BL/6 mice (five weeks old) obtained from the Animal Experiment Center at Huazhong Agricultural University (Wuhan, China) were used for the present study. In the experiments of NLRP3 in vivo, wild type and *NLRP3^-/-^* mice were generous obtained from were kindly obtained from Anding Zhang’s laboratory [40], mice were born in the same litter by heterozygotes (*NLRP3^+/-^*) mate with heterozygotes. The mice were housed under specific pathogen free conditions in an airconditioned room at 23±2^°^C. Food and water were supplied ad libitum. Animal welfare and experimental procedures were carried out in accordance with the criteria outlined in the National Institutes of Health Guide for the Care and Use of Laboratory Animals (NIH Publications No. 8023, revised 1978) and the related ethical regulations of Huazhong Agricultural University. All animal experimental protocols were approved by the Institutional Animal Care and Use Committee of Huazhong Agricultural University. All efforts were made to minimize animal suffering and to reduce the number of animals used. All animals were grouped according to their average weight.

### Establishment of DSS-induced mice colitis model and treatment

Acute colitis was induced by feeding the male C57BL/6 mice (five weeks old) with 3% (w/v) DSS dissolved in drinking water continuously for 7 days, mice in other groups drunk water without DSS. The experiment was randomly divided into five groups: control group, DSS group, and GPA (50, 100 or 150 mg/kg) + DSS groups. GPA (50, 100 or 150 mg/kg) was intragastrically administered for 7 days before and during DSS treatment via oral gavage once per day, GPA was dissolved in PBS, and mice in other groups were intragastrically administered for PBS. After 14 days, the mice were humanely euthanized, and the colons were excised, measured, and sectioned for further analysis (n=12/group).

Subsequently, to further confirm the key role of NLRP3 in the effect of GPA, GPA was intragastrically administered daily at 100 mg/kg. Male wild-type (WT) or NLRP3^-/-^ C57BL/6 mice (five weeks old) were randomly assigned to control group, DSS group, and GPA (100 mg/kg) + DSS group (n=10/group). Acute colitis was induced by feeding the male C57BL/6 mice (five weeks old) with 3% (w/v) DSS dissolved in drinking water continuously for 7 days, mice in other groups drunk water without DSS. GPA was intragastrically administered for 7 days before and during DSS treatment via oral gavage once per day, and mice in other groups were intragastrically administered for PBS.

### Evaluation of colitis severity

We evaluated the colitis severity on the basis of body weight, colon length, and macroscopic and microscopic observations of the stool and colon. The disease activity index (DAI) score was determined by the method reported in previous studies, with five grades of weight change (0, no weight loss or gain; 1, 1–5% loss; 2, 5–10% loss; 3, 10–20% loss; and 4, more than 20% loss), stool consistency (0, normal; 1, mild loose; 2, loose; 3, mild diarrhea; and 4, diarrhea), and stool bleeding (0, negative; 1, light bleeding; 2, mild bleeding; 3, severe bleeding; and 4, complete bleeding). Colon sections were prepared and stained with hematoxylin and eosin (H&E) according to standard protocols. All evaluation was blind.

### Cell death assay

Cell death was measured by PI incorporation. Cells were cultured in 12-well plates, after treatment, cells were stained with PI solution (2 μg/ml PI) for 10 min at room temperature, then washed three times using PBS, and observed immediately by live imaging using fluorescence microscope.

Cell death was measured by LDH release assay (Promega, Beijing, China), following the manufacturer’s instructions.

### ASC oligomerization assay and Semi-denaturing detergent agarose gel electrophoresis (SDD-AGE)

The oligomerization of ASC and NLRP3 were analyzed according to the published protocol [41, 42].

### The quantification of mtDNA by quantitative PCR

Total DNA was isolated from cells with DNA kit (TIANGEN, Beijing, China). The copy number of mtDNA was normalized to nuclear DNA (cytochrome c oxidase I/18S ribosomal RNA). The primers sequence was provided in the Supplementary Table 1.

### Measurement of ROS and mtROS production

The fluorescent probe DCFH_2_-DA (sigma) was used to detect the formation of intracellular ROS. After treatment, the cells were incubated with 10 μM DCFH_2_-DA at 37°C for 30 min. Finally, cells were washed with PBS, and the fluorescence was quantified on a FACS Calibur cytometry system (BD Biosciences) with excitation at 488 nm and emission at 530 nm. The results were expressed as percent of control Values.

The fluorescent probe mitoSOX (Invitrogen) was used to detect the formation of intracellular mtROS. After treatment, the cells were incubated with 5 μM mitoSOX at 37°C for 30 min. Finally, cells were washed with PBS, and the fluorescence was quantified on a FACS Calibur cytometry system (BD Biosciences) with excitation at 510 nm and emission at 580 nm. The results were expressed as percent of control Values.

### Intracellular potassium, chloride, or calcium detection

After treatment, the cells were collected, then intracellular potassium was detected by Potassium Assay Kit (Nanjing Jiancheng Bioengineering Institute, Nanjing, China), following the manufacturer’s instructions.

After treatment, the cells were collected, then the supernatants of 12-well plates were removed, ddH_2_O was added (200 μl/well), and the supernatants were kept 15 min at 37°C. The lysates were transferred to 1.5 ml EP tube, and centrifuged at 10,000 g for 5 min. 160 μl supernatants were then transferred to a new 1.5ml EP tube and mixed with 40 μl MQAE (10 μM) (Beyotime Biotechnology, Guangzhou, China). Absorbance was tested using BioTek Multi-Mode Microplate Readers.

After treatment, the supernatants of 12-well plates were removed, cells were with 4 μM Fluo-3AM (Beyotime Biotechnology, Guangzhou, China) for 30 min., then cells were washed by PBS for three times. Absorbance was tested using BioTek Multi-Mode Microplate Readers.

### Immunoblot analysis, immunoprecipitation (IP) and pull-down assay

The cells were extracted with protein lysis buffer (Beyotime, China) supplemented with protease inhibitor cocktail. The protein concentration was determined using the BCA Kit (Beyotime, China). Proteins (25–35 μg) were separated on a 10% polyacrylamide precast SDS gel (Bio-Rad, Richmond, CA, USA) followed by blotting on PVDF membranes (Millipore Billerica, MA, USA). For IP experiments, the cells were lysed in IP buffer (Beyotime, China) and incubated with IP-grade antibodies, followed by pull-down with protein A/G beads (161-4023) (Bio-Rad, Richmond, CA, USA) for subsequent immunoblot analyses.

For pull-down assay, THP-1 cell lysates were collected and centrifuged at 8000 × g. The supernatant was then transferred to another tube and the cell debris was thoroughly discarded. Prewashed streptavidin beads were added into the supernatant, allowing 2 h of preincubation with shaking at 4°C to remove unspecific binding proteins, followed by incubation with indicated doses of biotin-GPA for 6 h. Beads were washed with IP buffer for three times and boiled in SDS buffer.

### Immunofluorescence and confocal imaging

Cells were washed twice with PBS and fixed in 4% paraformaldehyde for 30 min at room temperature. 4,6-diamidino-2-phenylindole (DAPI) was used to label DNA. Confocal imaging was performed using a confocal laser scanning microscope (Carl Zeiss, Germany) equipped with an incubation chamber and a motorized table. Mitochondria were marked by MitoTracker (Red) (1:10 000 dilution) for 30 min before being fixed by 4% buffered formalin/PBS.

### ELISA

IL-1β in serum or cell supernatants were analyzed directly using an ELISA kit (NEOBIOSCIENCE, Shenzhen, China) according to the manufacturer’s instructions.

### Immunohistochemistry (IHC)

Immunohistochemical stains against MPO was detected using IHC kit (MaiXin, China). Briefly, paraffin embedded slides were deparaffinized, rehydrated, and washed in 1% PBS. Afterwards, they were incubated with 3% hydrogenperoxide and blocked with 10% goat serum for 1 h at 37°C. Then, the slides were treated with primary antibodies (1:100) overnight at 4°C. Biotinylated secondary anti-rabbit antibodies were added and incubated at room temperature for 1 h. Streptavidin-HRP was added, and after 40 min the sections were stained with a diaminobenzidine as a chromogen and counterstained with hematoxylin. Images at 200 × magnification were examined with a microscope (Olympus, Japan).

### Statistical analysis

No samples or animals were excluded from the analysis. Data were presented as the mean ± standard error. All data were accorded with normal distribution, and no variation was present. Differences between group means were determined by one-way ANOVA using SAS 8.0 software. The Tukey post hoc multiple comparison test was performed to compare significant variations. Differences were considered as significant at *P* < 0.05.

## Supplementary Material

Supplementary Figures

Supplementary Table 1
