# Supplementary material for: GPA peptide inhibits NLRP3 inflammasome activation to ameliorate colitis through AMPK pathway
Source: Aging (Albany NY). 2020 Sep 20;12(18):18522–44. doi: 10.18632/aging.103825 (PMC7585118; doi:10.18632/aging.103825)
Supplement: Supplementary Figures [file aging-12-103825-s002..pdf]

SUPPLEMENTARY FIGURES

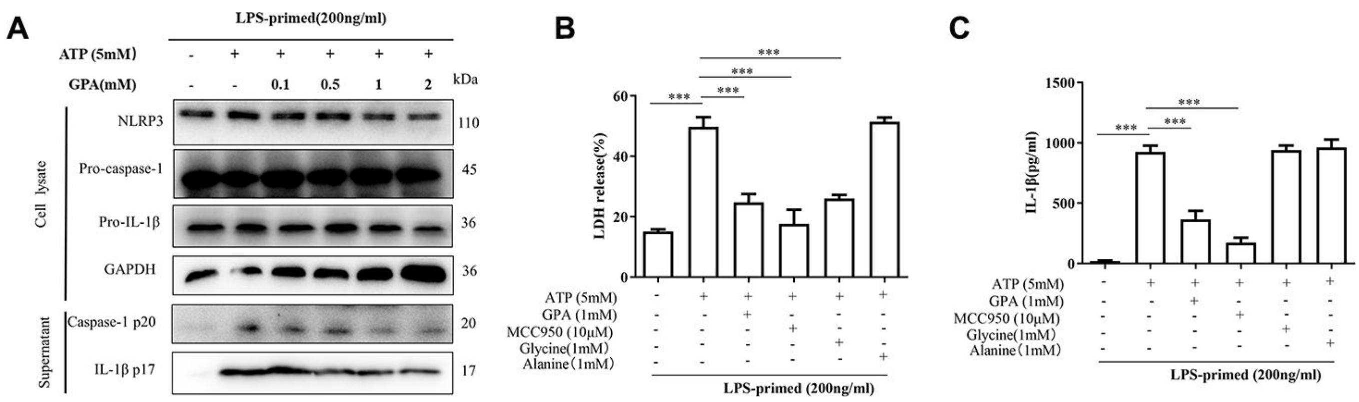

Supplementary Figure 1. GPA inhibited canonical NLRP3 inflammasome activation in BMDMs. BMDM cells were primed with LPS for 4 h, followed by GPA treatment 6 h before stimulation with ATP for 30 min. Immunoblot analyzed of IL-1β and caspase-1 in supernatants and cell lysate of BMDM cells (**A**). THP-1 cells were primed with LPS for 4 h, followed by GPA, MCC950, Glycine, and Alanine treatment 6 h before stimulation with ATP for 30 min. LDH and IL-1β in supernatants of THP-1 cells were detected (**B**, **C**). Data are presented as mean ± SD, three independent experiments. $^{*}p < 0.05$, $^{**}p < 0.01$ and $^{***}p < 0.001$.

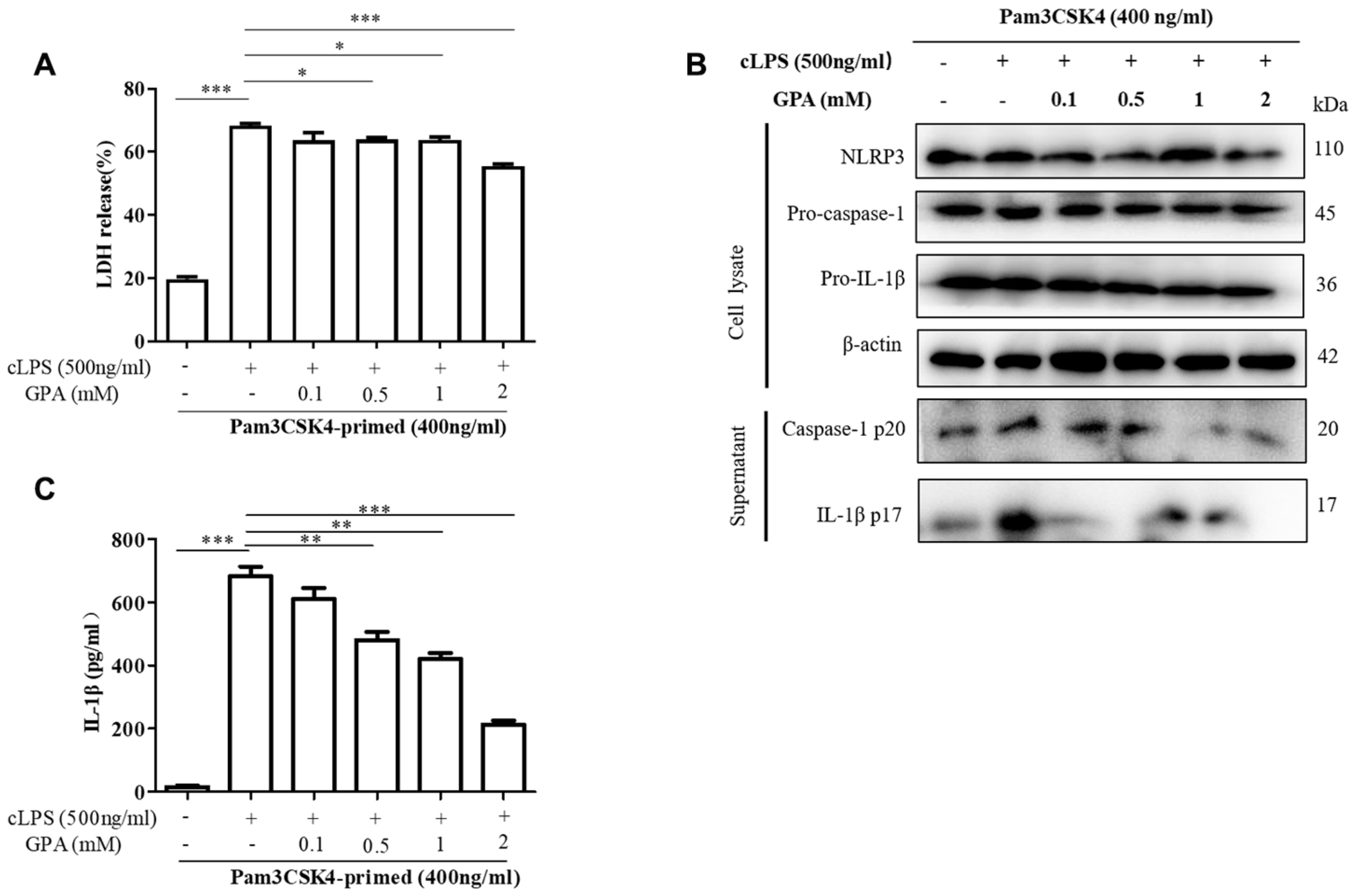

Supplementary Figure 2. GPA inhibited and non-canonical NLRP3 inflammasome activation in THP-1 cells. THP-1 cells were primed with Pam3CSK4 for 4 h, followed by GPA and cLPS treatment 16 h. Cell death was measured by and LDH released (**A**). Immunoblot analyzed of IL-1β and caspase-1 in supernatants and cell lysate of THP-1 cells (**B**). IL-1β in supernatants of THP-1 cells was detected by ELISA (**C**). Data are presented as mean ± SD, three independent experiments. $^{*}p < 0.05$, $^{**}p < 0.01$ and $^{***}p < 0.001$.

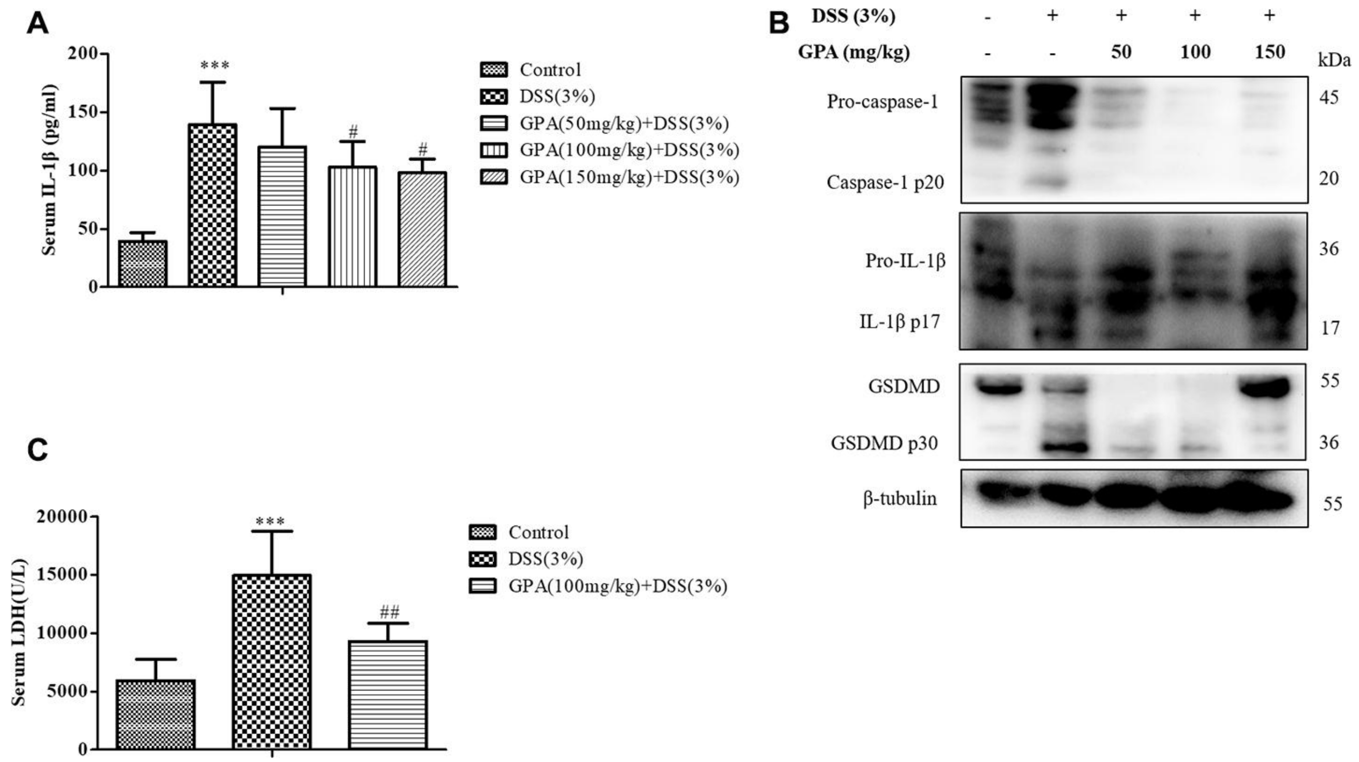

Supplementary Figure 3. GPA alleviated DSS-induced colitis in mice. IL-1β in serum of mice was detected by ELISA (**A**). Immunoblot analyzed of IL-1β, caspase-1 and GSDMD in colon tissues of mice (**B**). Cell death was measured by LDH released in serum (**C**). Data are presented as mean ± SD, n=6/group. $^{*}p < 0.05$, $^{**}p < 0.01$ and $^{***}p < 0.001$.

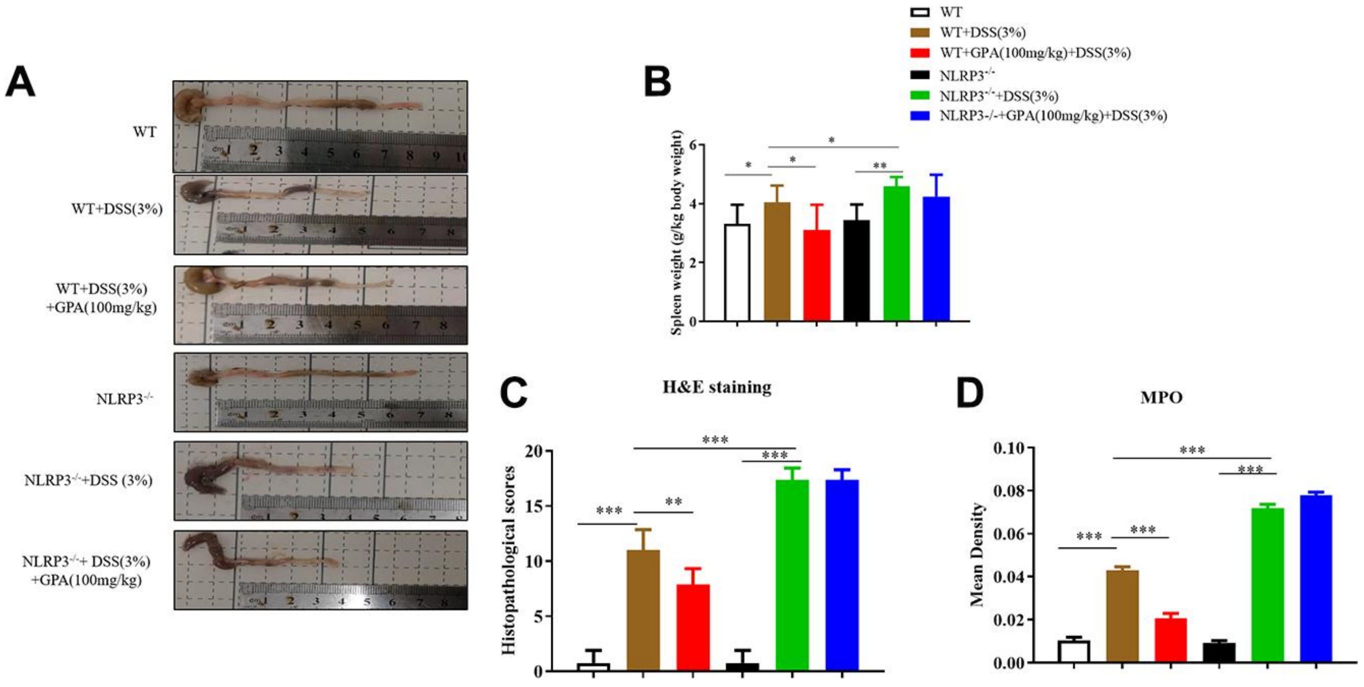

Supplementary Figure 4. GPA alleviated colitis depended on NLRP3. The lengths of colons from each group of mice (**A**). Spleen weights were measured (**B**). Analysis of histopathological scores in colon tissues by HE staining (**C**). Analysis of mean density of MPO in colon tissues by IHC (**D**). Data are presented as mean ± SD, n=10 /group. $^{*}p < 0.05$, $^{**}p < 0.01$ and $^{***}p < 0.001$.

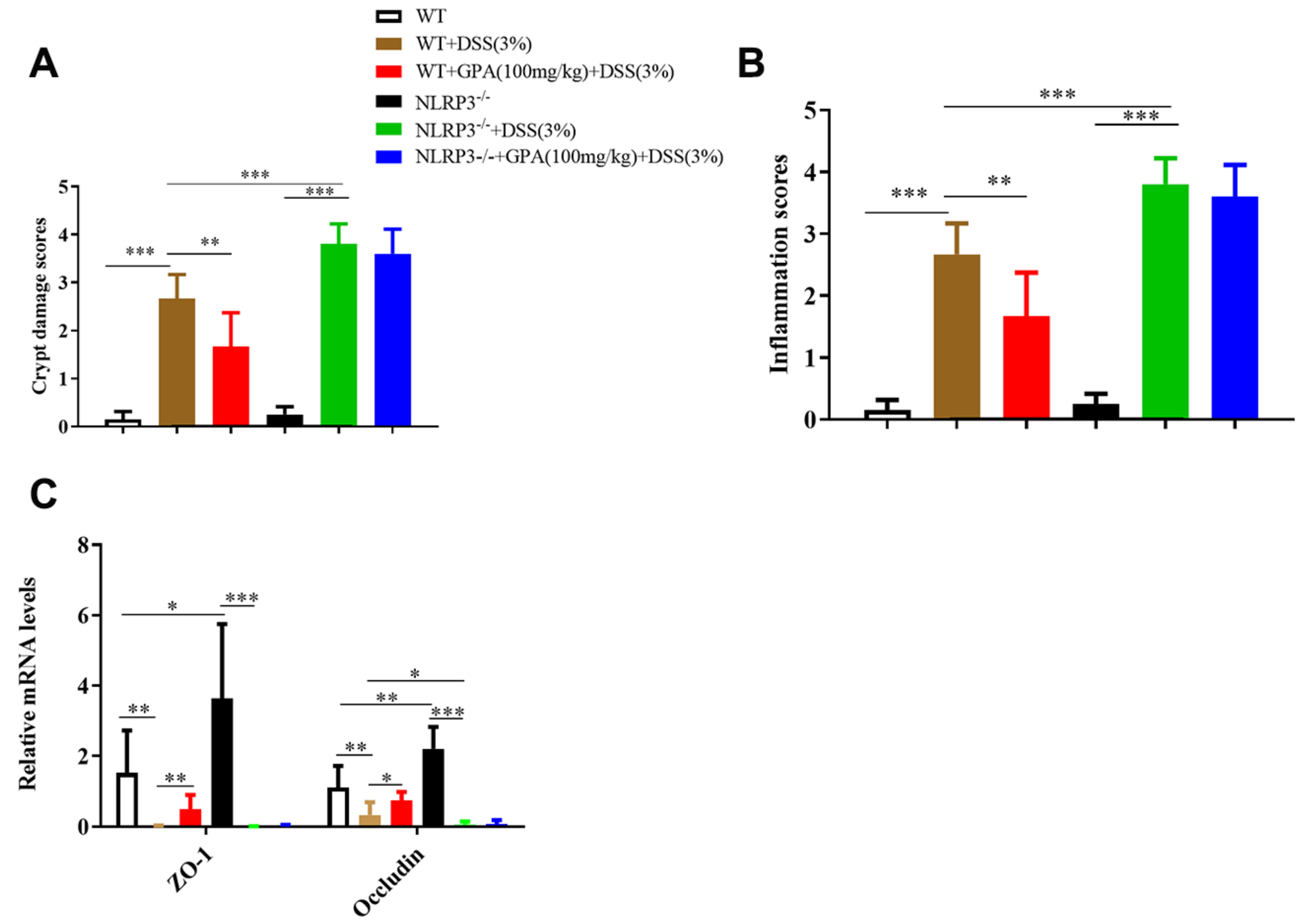

Supplementary Figure 5. GPA alleviated inflammation and maintained tight junction depended on NLRP3. Crypt damage and inflammation in colon tissues were evaluated by H&E stains (**A**, **B**). ZO-1 and Occludin mRNA levels in the colon tissues were measured by quantitative real-time PCR (**C**). Data are presented as mean ± SD, n=10 /group. $^{*}p < 0.05$, $^{**}p < 0.01$ and $^{***}p < 0.001$.

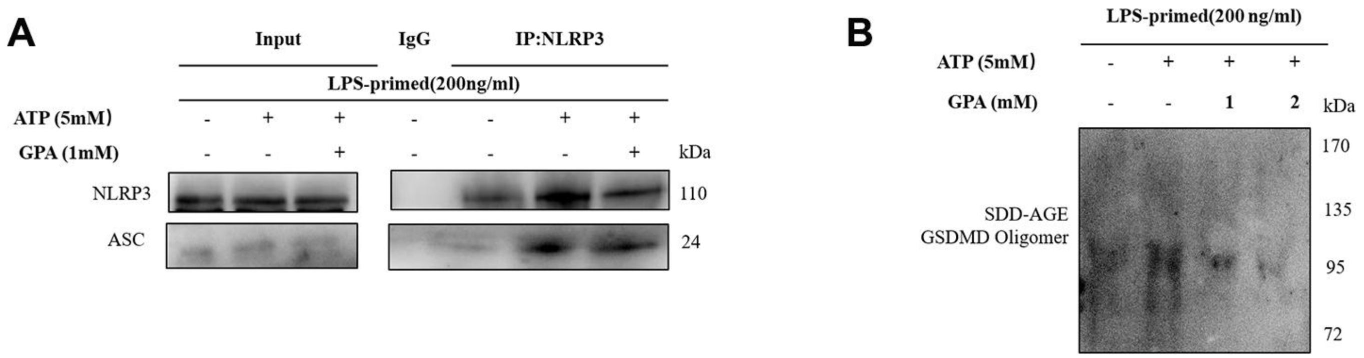

Supplementary Figure 6. GPA inhibited NLRP3 interaction with ASC, and oligomerization of GSDMD. THP-1 cells were primed with LPS for 4 h, followed by GPA treatment 6 h before stimulation with ATP for 30 min. IP and immunoblot analyzed of the interaction of endogenous NLRP3 and ASC in THP-1 cells (**A**). Immunoblot analyzed of GSDMD by SDD-AGE assay in THP-1 cells (**B**). Three independent experiments.

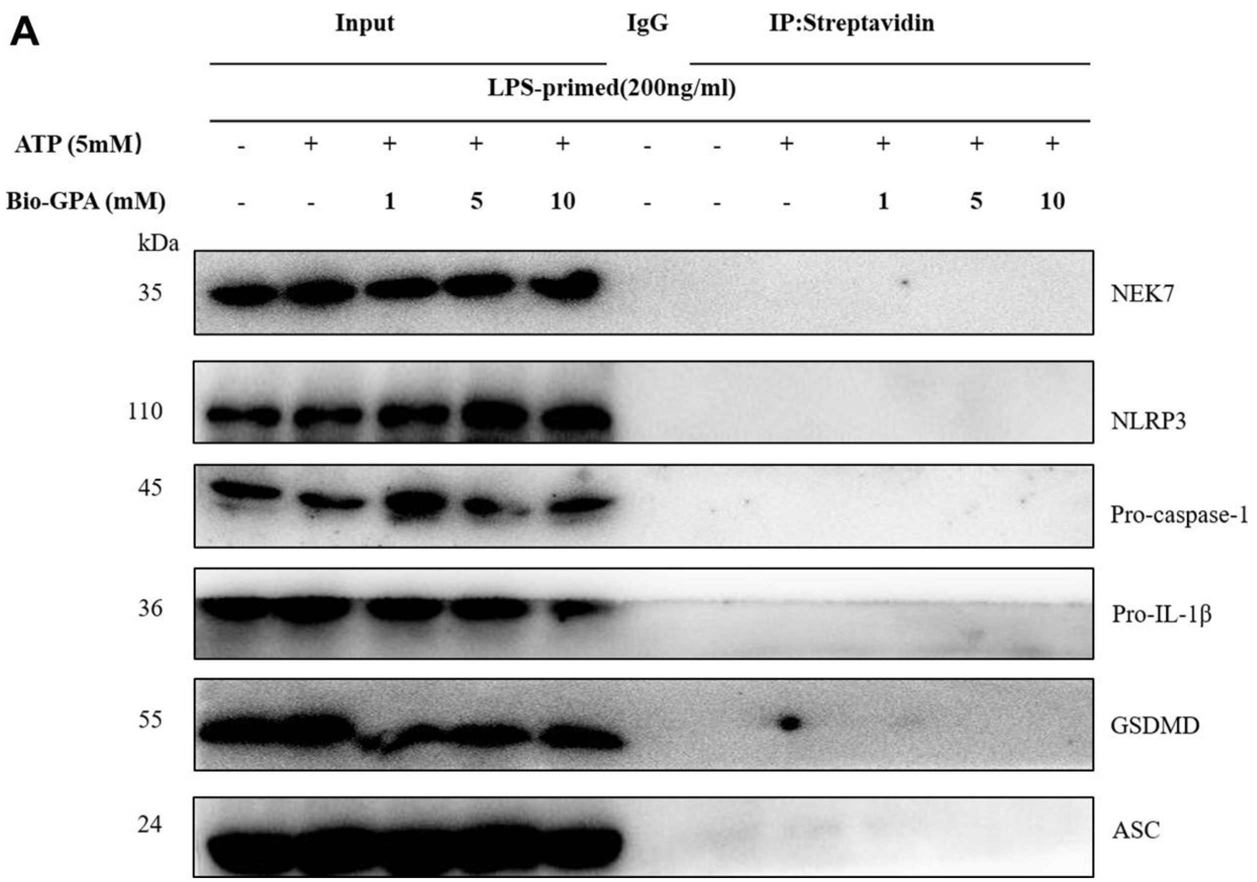

Supplementary Figure 7. Effect of GPA interaction with protein about NLRP3. THP-1 cells were primed with LPS for 4 h, followed by GPA treatment 6 h before stimulation with ATP for 30 min. Pull-down and immunoblot analyzed of the interaction of GPA and NLRP3 inflammasome in THP-1 cells (**A**). Three independent experiments.

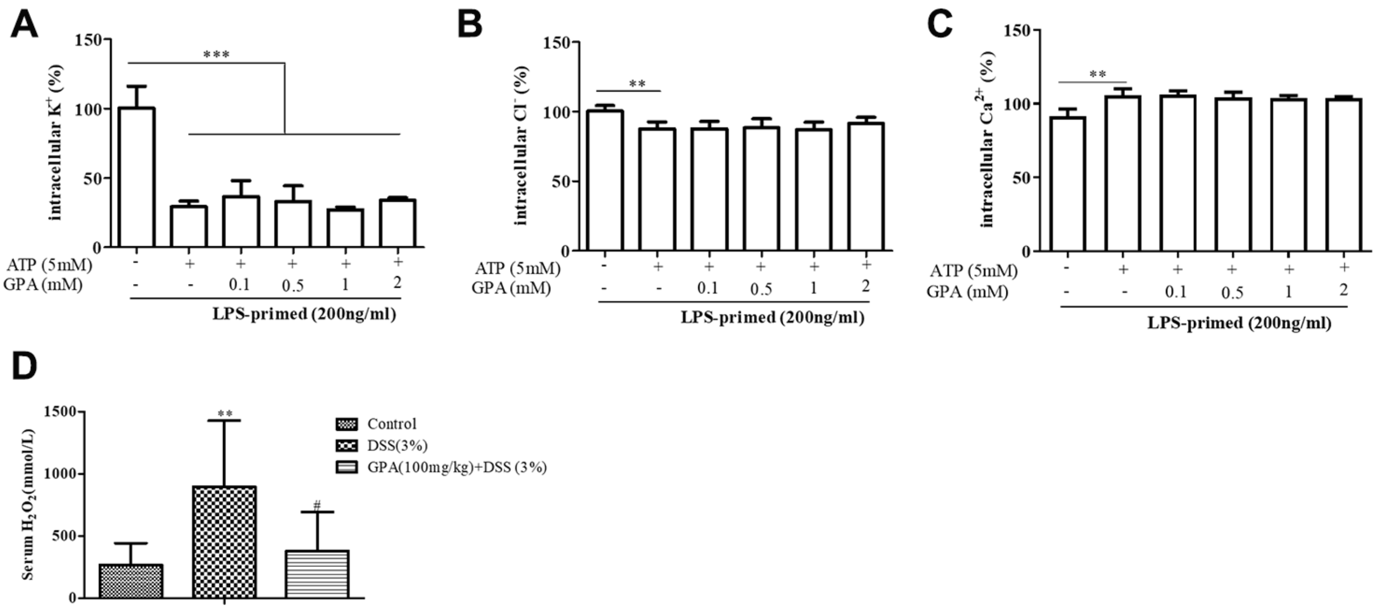

Supplmentary Figure 8. Effect of GPA on cell signals about NLRP3 inflammasome activation. Levels of the K^+, Cl^- and Ca^{2+} were measured in THP-1 cells (**A**–**C**). Data are presented as mean ± SD, three independent experiments. $^*p < 0.05$, $^{**}p < 0.01$ and $^{***}p < 0.001$. Oxidative stress was detected by level of H_2O_2 in serum (**D**). Data are presented as mean ± SD, n=12/group. $^*p < 0.05$, $^{**}p < 0.01$ and $^{***}p < 0.001$.

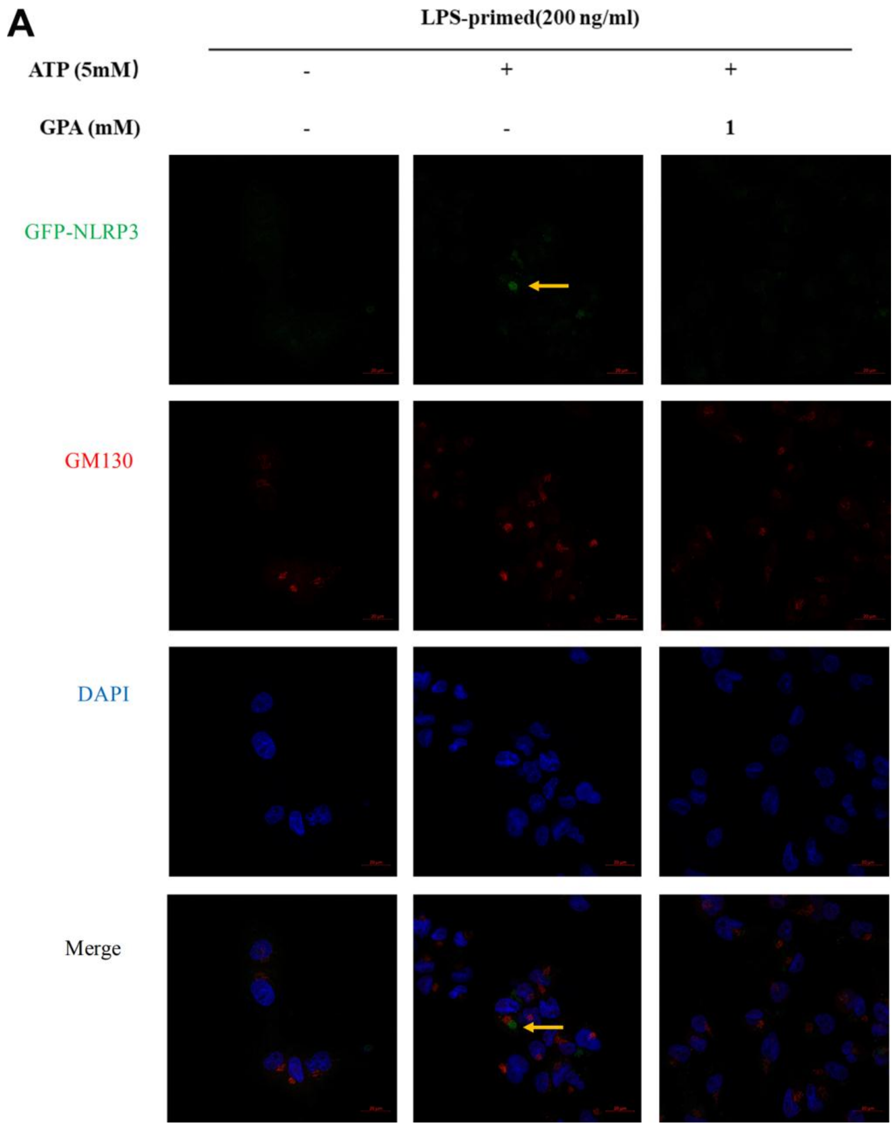

Supplementary Figure 9. Effect of GPA on NLRP3 Golgi localization. Immunofluorescence analyzed of Golgi components of NLRP3 in THP-1 cells (**A**). Three independent experiments.

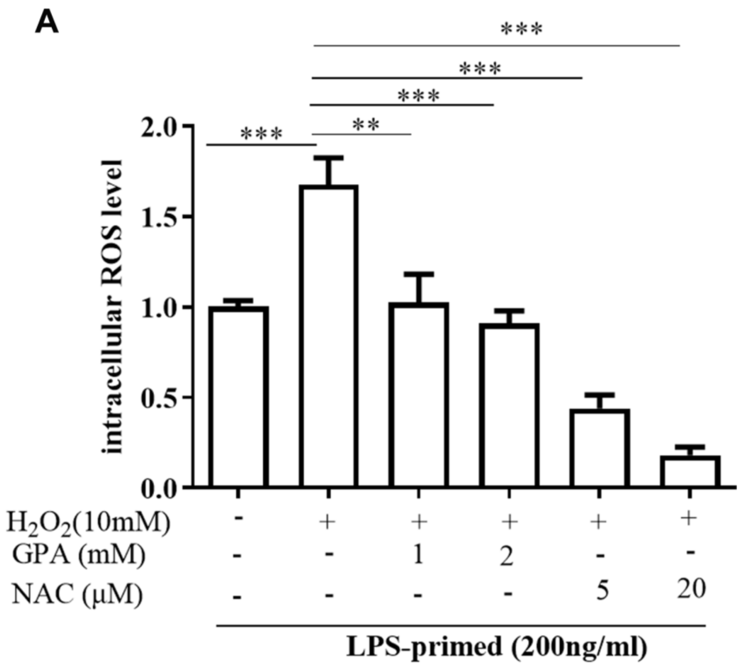

Supplementary Figure 10. GPA suppressed H_2O_2-induced ROS production. THP-1 cells were primed with LPS for 4h, followed by GPA or NAC treatment 6 h before stimulation with H_2O_2 for 4 h. Levels of the ROS was measured in THP-1 cells (**A**). Data are presented as mean ± SD, three independent experiments. $^{*}p < 0.05$, $^{**}p < 0.01$ and $^{***}p < 0.001$.

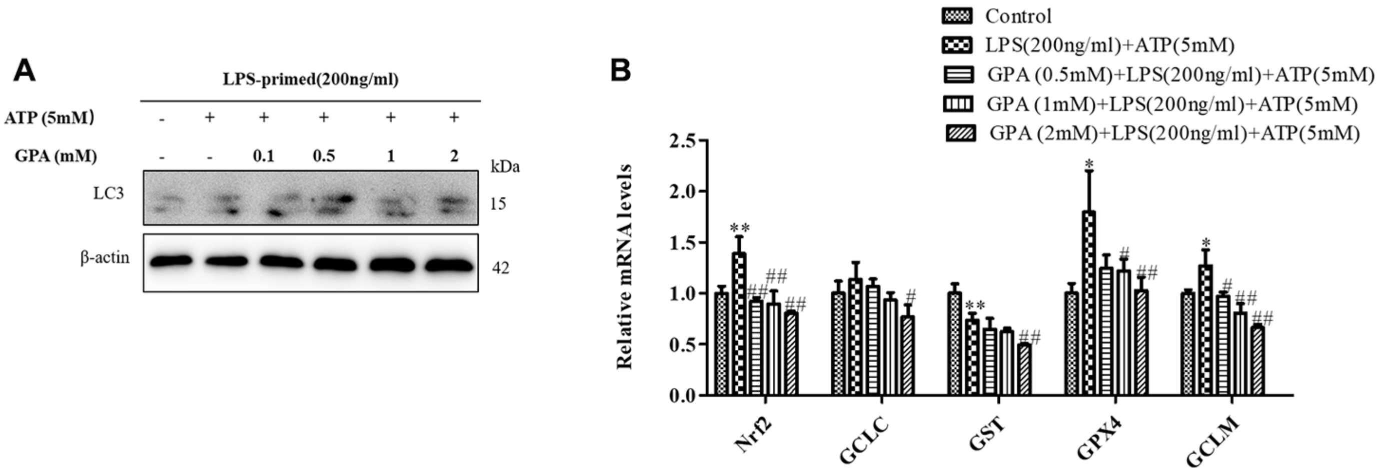

Supplementary Figure 11. Effect of GPA on autophagy and Nrf2. THP-1 cells were primed with LPS for 4 h, followed by GPA treatment 6 h before stimulation with ATP for 30 min. Immunoblot analyzed of LC3 in cell lysate of THP-1 cells (**A**). Nrf2, GCLC, GPX4 and GCLM mRNA levels in were measured by quantitative real-time PCR (**B**). Data are presented as mean ± SD, three independent experiments. $^{*}p < 0.05$, $^{**}p < 0.01$ and $^{***}p < 0.001$.
