# Supplementary material for: GPA peptide inhibits NLRP3 inflammasome activation to ameliorate colitis through AMPK pathway
Source: Aging (Albany NY). 2020 Sep 20;12(18):18522–44. doi: 10.18632/aging.103825 (PMC7585118; doi:10.18632/aging.103825)
Supplement: Supplementary Table 1 [file aging-12-103825-s001..pdf]

SUPPLEMENTARY TABLE

Supplementary Table 1. Primers used for RT-PCR analysis.

Primer	Primer sequences (5′ → 3′)	Product size (bp)
m-GAPDH	AACTTTGGCATTGTGGAAGG	132
	GGATGCAGGGATGATGTTCT	
m-ZO-1	TCATCCCAAATAAGAACAGAGC	198
	GAAGAACAACCCTTTCATAAGC	
m-Occludin	AAGCAAGTGAAGGGATCTGC	213
	GGGGTTATGGTCCAAAGTCA	
h-GAPDH	TCACCAGGGCTGCTTTTAACT	152
	GACAAGCTTCCCGTTCTCAG	
h-GPX-4	ACAAGAACGGCTGCGTGGTGAA<br>GCCACACACTTGTGGAGCTAGA	100
h-GCLM	TTGGAGTTGCACAGCTGGATT<br>TGGTTTTACCTGTGCCCACTG	200
h-GCLC	GCTGTCTTGCAGGGAATGTT<br>ACACACCTTCCTTCCCATTG	160
h-Nrf2	TCCAGTCAGAAACCAGTGGAT<br>GAATGTCTGCGCCAAAAGCTG	107
h-18S ribosomal RNA	ATCATGTTTGAGACCTTCAACA	318
	CATCTCTTGCTCGAAGTCCA	
h-cytochrome c oxidase I	CAAACCTACGCCAAAATCCA	164
	GAAATGAATGAGCCTACAGA	
